# Carboxylic Acid Fullerene (C_60_) Derivatives Attenuated Neuroinflammatory Responses by Modulating Mitochondrial Dynamics

**DOI:** 10.1186/s11671-015-0953-9

**Published:** 2015-05-30

**Authors:** Shefang Ye, Tong Zhou, Keman Cheng, Mingliang Chen, Yange Wang, Yuanqin Jiang, Peiyan Yang

**Affiliations:** Research Center of Biomedical Engineering, Department of Biomaterials, College of Materials, Xiamen University, Xiamen, 361005 People’s Republic of China; Department of Surgery, First Affiliated Hospital of Xiamen University, Xiamen, 361003 People’s Republic of China; Key Laboratory of Marine Biogenetic Resources, Third Institute of Oceanography, State Oceanic Administration, Xiamen, 361005 People’s Republic of China

**Keywords:** Fullerene derivatives, Mitochondria dynamics, Fission/fusion, Microglia, Neuroprotection

## Abstract

Fullerene (C_60_) derivatives, a unique class of compounds with potent antioxidant properties, have been reported to exert a wide variety of biological activities including neuroprotective properties. Mitochondrial dynamics are an important constituent of cellular quality control and function, and an imbalance of the dynamics eventually leads to mitochondria disruption and cell dysfunctions. This study aimed to assess the effects of carboxylic acid C_60_ derivatives (C_60_–COOH) on mitochondrial dynamics and elucidate its associated mechanisms in lipopolysaccharide (LPS)-stimulated BV-2 microglial cell model. Using a cell-based functional screening system labeled with DsRed2-mito in BV-2 cells, we showed that LPS stimulation led to excessive mitochondrial fission, increased mitochondrial localization of dynamin-related protein 1 (Drp1), both of which were markedly suppressed by C_60_–COOH pretreatment. LPS-induced mitochondria reactive oxygen species (ROS) generation and collapse of mitochondrial membrane potential (Δ*Ψ*m) were also significantly inhibited by C_60_–COOH. Moreover, we also found that C_60_–COOH pretreatment resulted in the attenuation of LPS-mediated activation of nuclear factor (NF)-κB and mitogen-activated protein kinase (MAPK) signaling, as well as the production of pro-inflammatory mediators. Taken together, these findings demonstrated that carboxylic acid C_60_ derivatives may exert neuroprotective effects through regulating mitochondrial dynamics and functions in microglial cells, thus providing novel insights into the mechanisms of the neuroprotective properties of carboxylic acid C_60_ derivatives.

## Background

Mitochondria are organized in a highly dynamic tubular network that is continuously shaped by complementary fission and fusion events [1]. Mitochondria dynamics regulate processes associated with mitochondria morphology such as mitochondria biogenesis, localization, and distribution as well as the morphology itself [2]. Defects in either fission or fusion limit mitochondrial motility, decrease energy production, and promote oxidative stress, thereby resulting in cell dysfunction and death [3]. Mitochondrial fission and fusion processes are regulated by evolutionarily conserved molecular machinery. In mammalian cells, a core machinery of mitochondria-shaping proteins exists that impinges on the fusion-fission equilibrium. It has been reported that the large GTPase proteins, including outer membrane mitofusins (Mfn-1 and Mfn-2) and inner membrane optic atrophy 1 (Opa1), assist in the mitochondrial fusion process in mammalian cells [4]. Another class of GTPase proteins including dynamin-related protein 1 (Drp1) promote mitochondrial fission by interacting with its adaptor in the outer membrane such as fission protein 1 (Fis1) and mitochondrial fission factor [5]. Imbalanced mitochondrial dynamics are directly linked to many human diseases including neurodegenerative diseases, metabolic disorders, and cancer [6]. Despite accumulating data currently available regarding the machinery of mitochondrial fission and fusion, the precise molecular mechanisms are still not fully understood. Identification of regulator of mitochondria dynamics should lead to the development of new therapeutic strategies for treating mitochondria-associated diseases [4–6].

Fullerene C_60_, also called as buckminsterfullerenes or buckyballs, is a unique spherical carbon molecule [7]. Due to its highly unsaturated structure and excellent electron-receptor properties, C_60_ has been extensively investigated as a radical scavenger [8]. This property makes them attractive therapeutic options for the treatment of oxidative stress-related chronic disorders. However, the extreme hydrophobicity and potential toxicity of fullerene limit its application as a therapeutic agent. Derivatization of water-soluble fullerenes by directly adding hydrophilic group (eg., coupling with –COOH, −OH, or –NH_2_) moieties to the carbon cage has been used as a strategy to produce useful drug candidates [9], and the evidence clearly demonstrated that with increasing hydrophilicity, their toxicity decreased [7, 8]. Such C_60_ derivatives including polyhydroxylated C_60_ (fullerenol) [10, 11], carboxylated fullerenes [10], and polysulfonated C_60_ [12] have been shown to be useful for a wide range of biomedical applications, including drug and gene delivery [13], DNA photocleaving [7], extinction of reactive oxygen species (ROS) [10, 11, 14], anti-apoptosis [15, 16], antiviral activity [17], photodynamic therapy [7, 10], neuroprotection [18–20], anti-inflammation [21], and modulation of learning and memory [22]. Recently, the anti-allergic [23], tumor-inhibitory, and immunomodulatory properties [24, 25] of fullerene derivatives have also been described.

Microglia, the resident immune cells of the central nervous system (CNS), participate in both innate and adaptive immune responses [26]. However, overactivated microglia cells are able to exert detrimental neurotoxic effects through the excessive production of various toxic factors, such as chemokines, eicosanoids, cytokines, and reactive free radicals [27]. Although these factors are necessary for immune surveillance of the local brain environment, microglial responses must be properly and tightly regulated so as to avoid overactivation and associated disastrous neurotoxic consequences. Thus, inhibition of microglial activation and subsequent inflammatory process may provide therapeutic benefits for neurodegenerative disorders [28]. Regarding their various biological and pharmacological properties, water-soluble carboxylic acid C_60_ derivatives have been extensively investigated in the prevention of neurodegenerative diseases, though molecular details are still largely lacking. In this study, we sought to examine the effects of carboxylic acid C_60_ derivatives (C_60_–COOH) on the neuroninflammatory response in lipopolysaccharide (LPS)-stimulated microglial BV-2 cell model, and elucidate whether modulation of mitochondrial dynamics are related to C_60_–COOH-mediated attenuation of inflammatory responses.

## Methods

### Preparation of Carboxylic Acid C_60_ Derivative

**Fig. 1:**
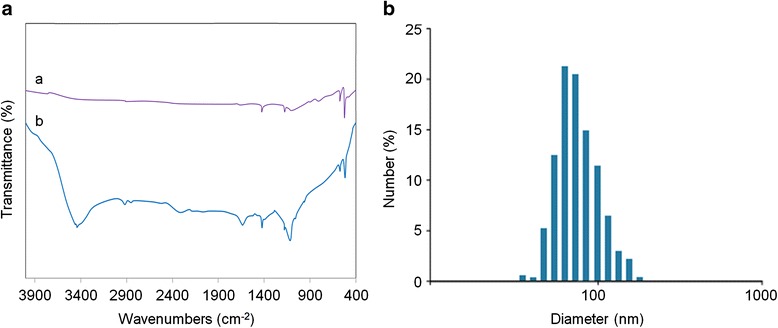
**a** FTIR spectrum of (*a*) raw C_60_; (*b*) C_60_(C(COOH)_2_)_3_. **b** The size distribution of C_60_(C(COOH)_2_)_3_ in culture medium (10 % fetal bovine serum) obtained by dynamic light scattering (DLS). The concentration of C_60_(C(COOH)_2_)_3_ was 50 μM

A water-soluble carboxylic acid C_60_ derivative was prepared according to a previously reported method [29]. In brief, NaH was added to the solution of C_60_ (99.5 % pure; Sigma, St. Louis, MO) in toluene. After the solution changed to a dark-red color, diethyl bromomalonate (Sigma, St Louis, MO, USA) was added. Under the protection of argon, the solution was stirred at 25 °C for 10 h. After removal of the liquid phase, the residue was eluted by toluene followed by addition of excess NaH. The solution was stirred under argon atmosphere at 80 °C for 10 h, and the reaction was terminated by addition of methanol containing HCl. The precipitate was then collected by centrifugation, washed thoroughly with methanol, HCl, and deionized water, and then subjected to silica gel column chromatography. MALDI-TOF mass spectrometry (Shimadzu Biotech, Kyoto, Japan) verified the targeting product (m/z = 1107). The Fourier transform infrared spectroscopy (FTIR; iN10 MX IR, Nicolet) spectra exhibits main peaks at 3439, 1718, 1201, and 523 cm^−1^, confirming that C_60_ have been successfully modified by –COOH (Fig. 1a). The molecular formula of carboxylic acid C_60_ derivative was established as C_60_(C(COOH)2)_3_ (C_60_–COOH for short). Dynamic light scattering (DLS) data using a Zetasizer Nano ZS (Malvern Instruments, Malvern, UK) showed that the C_60_–COOH (50 μM) in culture medium (10 % fetal bovine serum) appeared homogeneous with the average size 82.5 nm (Fig. 1b). The C_60_–COOH suspension were shown to be negative for endotoxins using the limulus amebocyte lysate test (Pyrogent 5000®; Lonza, Walkersville, MD).

### Cell Culture and DNA Transfection

The murine BV-2 microglial cell lines (Cell Bank of Chinese Academy of Sciences, Shanghai, China) were grown in Dulbecco’s modified Eagle’s medium (DMEM; Gibco, GrandIsland, NY, USA) supplemented with 10 % heat-inactivated fetal bovine serum (FBS; Life Technologies, Carlsbad, CA, USA), streptomycin (10 μg/mL), and penicillin (10 U/ml) (Invitrogen, Carlsbad, CA), and maintained at 37 °C in a humidified 5 % CO_2_ incubator. The cells were grown to a density of 2 × 10^5^ in a six-well plate for 24 h at 37 °C prior to initiating the experiments.

The DsRed2-mito gene, which contains DNA sequences coding for DsRed2 fused at the 3′ end of the mitochondrial targeting sequence from subunit VIII of cytochrome c oxidase, was derived from the plasmid pDsRed2-Mito (Clontech, Palo Alto, CA, USA). The DsRed2-mito coding sequence was amplified by PCR using LA Taq polymerase (Takara, Shiga, Japan), and sequentially cloned into a lentiviral vector pLenti6/V5-DEST (Invitrogen, Carlsbad, CA, USA), which contains a c-terminal V5 epitope tag that can be fused to a gene of interest to allow protein detection by immunoblotting. For transfection study, DsRed2-mito lentiviral vectors at multiplicity of infection (MOI) of 5:1 were treated with 8 μg/mL polybrene (Sigma, St Louis, MO, USA). The cells were then cultured for 72 h and the DsRed2-mito expressing BV-2 cells were then selected for resistance to 4 μg/mL blasticidin (Invitrogen, Carlsbad, CA, USA). The resulting cell colonies were used for subsequent proliferation and storage.

### Cell Viability Assay

Cell viability was determined using 3-(4,5-dimethylthiazol-2-yl)-2,5-diphenyltetrazolium bromide (MTT; Sigma, St Louis, MO, USA) assay as previously described [11]. Briefly, BV-2 cells were grown on 96-well plates at a density of 5 × 10^4^ for 24 h. After treatment with C_60_–COOH at various doses (10–100 μM) for 24 h, the cells were incubated with MTT solution (0.5 mg/mL, 1 × PBS) for 2 h at 37 °C. The formazan crystals resulting from mitochondrial enzymatic activity on MTT substrate were solubilized with 200 μL of dimethyl sulfoxide, and absorbance at 570 nm was measured using a microplate reader (Model 680, Bio-Rad Laboratories, Hercules, CA, USA). The results are given relative to the untreated control.

### Analysis of Mitochondrial Morphology

BV-2 cells expressing DsRed2-mito were seeded on poly-D-lysine coated glass. The cells were treated with 1 μg/mL LPS (Sigma, St. Louis, MO) for 12 h with or without 50 μM C_60_–COOH pretreatment for 6 h. Cells were fixed with 4 % paraformaldehyde for 15 min and then incubated with 0.5 μM MitoTracker green (Invitrogen, Carlsbad, CA, USA) at 37 °C for 15 min. Images of the cells were acquired using an LSM-710 confocal microscope (Carl Zeiss, Oberkochen, Germany). Mitochondrial length was measured with ImageJ software (NIH, Bethesda, MD, USA). Analysis of mitochondrial morphology was performed by counting more than 50 mitochondrial particles per cell in over 20 cells according to a method previously described [30]. Mitochondria were divided into different categories based on length ranging from less than 1 μm, 1–3 μm, and greater than 3 μm.

### Measurement of Mitochondrial ROS Production

BV-2 cells were seeded at a density of 5 × 10^4^ cells in each well of 96-well plates and allowed to attach over night, and then incubated with 1 μg/mL LPS for 6 h with or without 50 μM C_60_–COOH pretreatment for 6 h. To examine accumulation of mitochondrial superoxide, trypsinized BV-2 cells were incubated with 2.5 μM MitoSOX Red mitochondrial superoxide indicator (Invitrogen, Carlsbad, CA) 37 °C for 30 min, washed twice with phosphate-buffered saline (PBS), and centrifuged at 800 g for 5 min. Cell pellets were re-suspended in ice-cold PBS and quantification of MitoSOX Red fluorescence was analyzed using a FACScan flow cytometry (BD Biosciences) with excitation and emission wavelengths at 510/580 nm.

### Mitochondrial Membrane Potential (Δ*Ψ*m) Assay

The lipophilic cationic probe 5,5′,6,6′-tetrachloro-1,1′,3,3′- tetraethylbenzinidazolylcarbocyanine iodide (JC-1) dye (Molecular Probes, Eugene, OR) was used to measure mitochondrial inner membrane potential (Δ*Ψ*m) according to the manufacturer’s instructions. JC-1 accumulates in the mitochondria in proportion to Δ*Ψ*m and forms aggregates emitting red fluorescence. In the cytoplasm, JC-1 exists as monomers emitting red fluorescence. Briefly, after incubation with 1 μg/mL LPS for 6 h in the absence or presence of 50 μM C_60_–COOH pretreatment for 6 h, cells were trypsinized and incubated with 5 μg/mL JC-1 for 30 min at room temperature in the dark. Then, the cells were washed twice with PBS and visualized under a fluorescence microscope (Eclipse TE300, Nikon, Japan).

### Immunofluorescence Staining

BV-2 cells were fixed with 4 % paraformaldehyde at room temperature for 20 min, permeabilized with 0.5 % Triton X-100 in PBS, and then incubated with blocking buffer (PBS, 5 % goat serum, and 0.3 % Triton X-100) for 30 min. The cells were then labeled with primary antibodies in blocking buffer at 4 °C overnight, followed by incubation with secondary antibody. Thereafter, cells were nuclear-stained via 15-min incubation in a blocking solution containing 0.25 mg/mL DAPI (Santa Cruz, CA, USA). Fluorescent-labeled cells were imaged with LSM-710 confocal microscope (Carl Zeiss, Oberkochen, Germany).

### Protein Extract

Harvested BV-2 cells were washed twice with ice-cold PBS. Then, the cells were lysed on ice in lysis buffer consisting of 50 mM Tris–HCl (pH 8.0), 150 mM NaCl, 0. % sodium dodecyl sulfate (SDS), 1 % Triton X-100, 0.5 % sodium deoxycholate, 1 mM EDTA (Sigma, St. Louis, MO, USA), 1 % protease inhibitor cocktail, and 1 % phosphatase inhibitor cocktail (Roche Diagnostics Co., Indianapolis, IN). Nuclear and cytoplasmic fractions were isolated using an NE-PER nuclear and cytoplasmic extraction reagent kit (Thermo Scientific, Waltham, MA, USA). Mitochondrial fractions were prepared with a mitochondria isolation kit according to the manufacturer’s protocol (Thermo Scientific, Waltham, MA, USA). The protein concentration was determined using the protein assay reagent (Bio-Rad Laboratories, Hercules, CA).

### Western Blot Analysis

Equal amounts of whole cell protein as well as nuclear, mitochondrial, and cytoplasmic lysates were separated by electrophoresis in 8–12 % sodium dodecyl sulfate polyacrylamide (SDS-PAGE) gels and transferred onto nitrocellulose membranes (Millipore, Bedford, MA, USA). Primary antibodies used for immunoblotting included anti-Drp1, anti-phosphorylated(p)-Drp1(Ser637), anti-COX IV, anti-ERK, anti-p-ERK, anti-JNK, anti-p-JNK, anti-p38, anti-p-p38 (Santa Cruz, CA, USA), anti-iNOS, anti-COX-2, anti-β-actin, anti-Mfn1, anti-Mfn2, anti-Fis1 (Sigma, St Louis, MO, USA), anti-Opa1 (BD Biosciences), anti-V5, anti-NF-κB p65, and anti-lamin B (Abcam, Cambridge, MA, USA). Membranes were incubated overnight at 4 °C with primary antibodies and then with their corresponding horseradish peroxidase-conjugated secondary antibodies for 1 h at room temperature. The immunoblots were detected with enhanced chemiluminescence (ECL) reagents (Amersham Biosciences, Piscataway, NJ, USA). Densitometry data analysis was performed using ImageJ software (NIH, Bethesda, MD, USA) and expressed in arbitrary units.

### Measurement of Inflammatory Mediators

BV-2 cells were pretreated with 50 μM C_60_–COOH for 6 h and then stimulated with 1 μg/mL LPS for 12 h. Subsequently, prostaglandin E2 (PGE_2_), tumor necrosis factors-α (TNF-α), interleukin-1β (IL-1β), and IL-6 levels were quantified in the culture supernatants using enzyme-linked immunosorbent assay (ELISA) kit (R&D Systems, Minneapolis, MN), and nitric oxide (NO) production was assayed using the Griess reagent assay (Invitrogen, Carlsbad, CA, USA) according to the manufacturer’s instructions.

### Statistical Analysis

Results are presented as the means ± standard deviation of the triplicate experiments. Comparisons between groups were evaluated by two-sided Student’s *t* test or one-way analysis of variance. A *p* value of less than 0.05 was considered statistically significant.

## Results and Discussion

### The Effects of C_60_–COOH on LPS-Induced Mitochondrial Fission

**Fig. 2:**
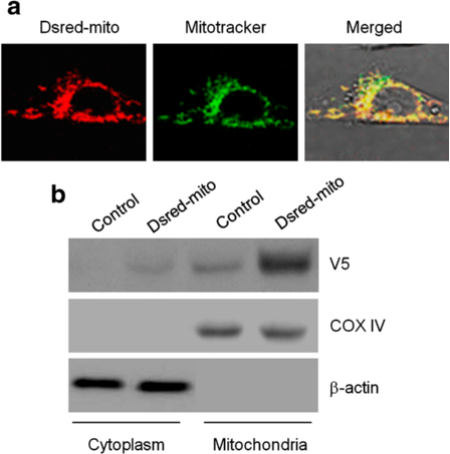
**a** BV-2 cells expressing the DsRed2-mito were observed with a laser confocal microscopy after staining with MitoTracker green. **b** Cytoplasm and mitochondrial fractions of wild-type and DsRed2-mito BV-2 cells were analyzed by western blotting with antibodies against theV5-epitope. Cytochrome c oxidase subunit IV (COX IV) and β-actin were used as a mitochondria and cytoplasm marker, respectively

To observe mitochondrial morphological changes, BV-2 cells were transiently transfected with a lentiviral vector encoding mitochondria-targeting DsRed2 (DsRed2-mito) [31]. Confocal laser microscopy confirmed that the expression of DsRed2-mito gene exhibited a characteristic punctuate pattern of staining (Fig. 2a). To verify this finding, the cells were co-stained with mitochondrial-specific staining dyes Mitotracker green. Superimposition of the two images revealed a considerable degree of overlap between endogenous DsRed2-mito staining and the mitochondrial staining (Fig. 2a). Western blotting analysis of mitochondrial and cytosolic fractions confirmed that the expression of DsRed2-mito protein was only observed in the mitochondrial fraction in BV-2 cells with a V5 antibody (Fig. 2b).

**Fig. 3:**
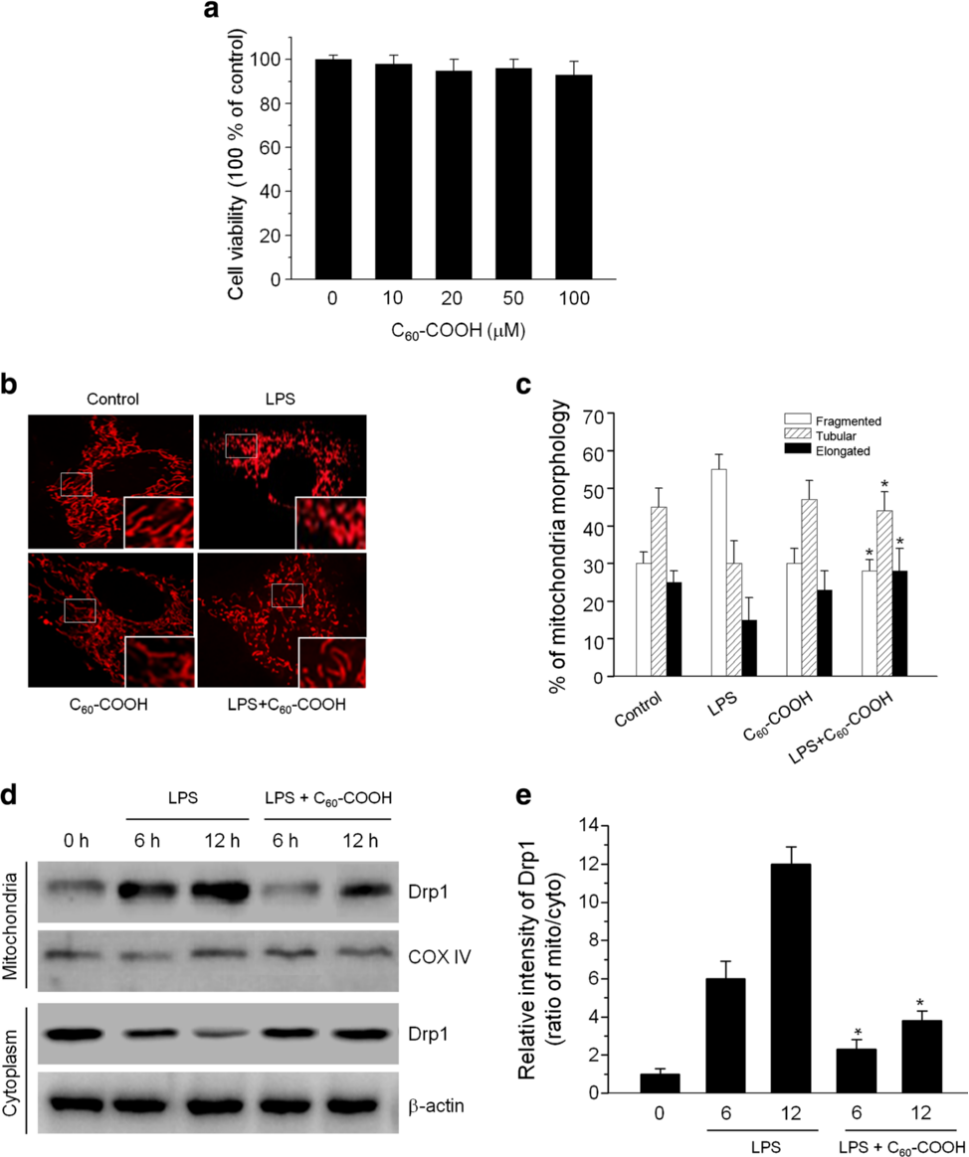
**a** BV-2 cells were incubated with increasing doses of C_60_–COOH (10–100 μM) for 24 h, and the cell viability was determined by MTT assay. The data are expressed as the mean ± SD of three independent experiments. **b** The effect of C_60_–COOH on LPS-induced mitochondrial fragmentation. BV-2 cells transfected with DNA encoding DsRed2-mito were treated with 1 μg/mL LPS for 12 h in the presence or absence of with 50 μM C_60_–COOH pretreatment for 6 h. Mitochondrial morphology was observed under a laser confocal microscope. Representative images are shown. Magnified views (fragments) are shown in *insets*. **c** Graphs present data from the analysis of mitochondria morphology using ImageJ software. The results are for at least 20 cells per condition in each experiment. **d** BV-2 cells were stimulated with 1 μg/mL LPS for 6 or 12 h in the presence or absence of 50 μM C_60_–COOH pretreatment for 6 h, then the cytoplasmic and mitochondrial fractions were isolated and translocation of Drp1 was analyzed by western blotting using antibodies against Drp1. COX IV and β-actin were served as mitochondria and cytoplasmic markers, respectively. **e** The ratio of Drp1 expression in mitochondria and cytoplasm were performed by densitometric analysis. The data are expressed as the mean ± SD of three independent experiments. *p < 0.05, significantly different from LPS-treated control

In healthy cells, mitochondrial fusion and fission is a dynamic process critical for the maintenance of mitochondrial function and cell viability [2]. The change in fission/fusion balance impacts mitochondrial function, and LPS has been shown to affect this dynamics by upregulating the fission protein dynamin-related protein 1 (Drp1), which results in disrupted distribution and fragmentation of mitochondria [32]. In order to determine the optimal concentration of C_60_–COOH for following analysis, we initially screen the sensitivity of BV-2 cells to C_60_–COOH. Previous reports indicated that carbon-based nanomatetials such as single-walled carbon nanotubes (SWCNTs) might interfere when tested with MTT [33]. However, MTT is a widely accepted test method for in vitro toxic study of C_60_ derivatives [19, 34]. Results obtained from MTT assays showed that C_60_–COOH up to 100 μM for 24 h was well tolerated by BV-2 cells without any influence on cell viability (Fig. 3a). For comparison to previous work on C_60_–COOH in vitro studies [14, 16], a dose of 50 μM C_60_–COOH was used in the subsequent experiments. To examine the effects of C_60_–COOH on mitochondrial morphological changes, we established a cell-based functional screening system using BV-2 cells that stably expressed the DsRed2-mito gene. DsRed2-mito expressing BV-2 cells were treated with 1 μg/mL LPS in the absence or presence of 50 μM C_60_–COOH pretreatment for 12 h, and the mitochondrial network was visualized by confocal laser microscopy. As shown in Fig. 3b, characteristics of mitochondrial fragmentations such as punctate and shorter mitochondria in LPS-stimulated DsRed2-mito BV-2 cells were clearly visible after 12 h compared to the untreated cells, in agreement with earlier reports [32]. However, C_60_–COOH pretreatment significantly inhibited mitochondrial fragmentations induced by LPS in BV-2 cells (*p* < 0.05) (Fig. 3c).

### C_60_–COOH Inhibited LPS-Induced Translocation of Drp1 to the Mitochondria

**Fig. 4:**
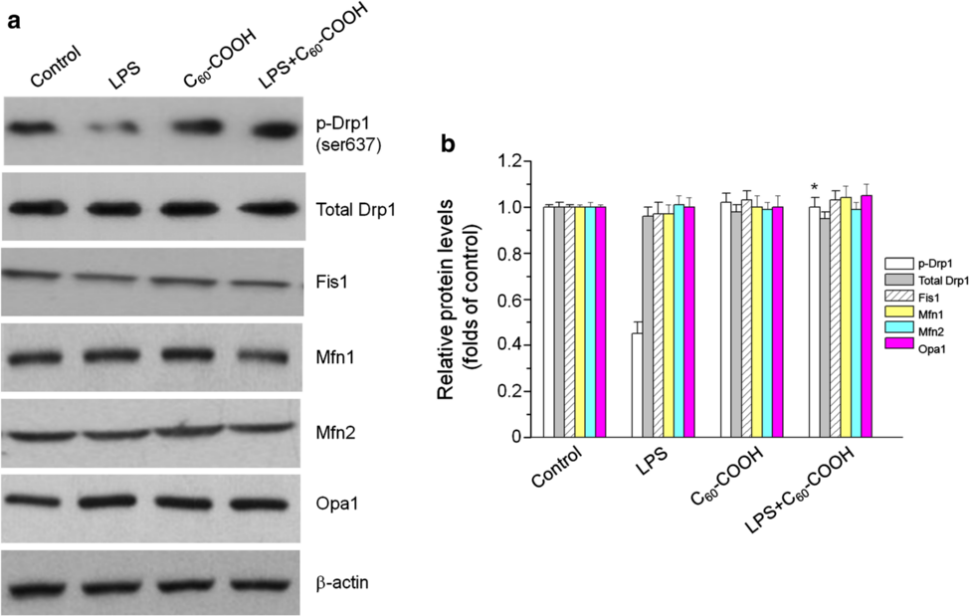
**a** Western blotting analysis of fission proteins (Fis1) and fusion proteins [Drp1, p-Drp1(ser637), Mfn1, Mfn2, and Opa1] in BV-2 cells stimulated with 1 μg/mL LPS LPS for 12 h in the presence or absence of 50 μM C_60_–COOH pretreatment for 6 h was performed. **b** The relative levels of fission proteins and fusion proteins expression in mitochondria and cytoplasm were performed by densitometric analysis. The data are expressed as the mean ± SD of three independent experiments. *p < 0.05, significantly different from LPS-treated control

BV-2 cells pretreated with C_60_–COOH exhibited less mitochondrial fragmentation compared with LPS-stimulated cells, which suggested that C_60_–COOH represent a novel inhibitor of mitochondrial fragmentation. Next, we measured the levels of mitochondrial fission and fusion proteins as evidence of mitochondrial fragmentation. It has been reported that mitochondrial fission requires translocation of Drp1 from the cytosol to mitochondria. In addition, translocation of Drp1 to mitochondria is controlled by Serine637, and phosphorylation of Drp1 at Serine637 leads to mitochondria fusion, while de-phosphorylation promotes mitochondrial fission [35]. Western blotting analysis of subcellular fractions revealed that LPS induced translocation of a significant amount of Drp1 to the mitochondria in a time-dependent manner (Fig. 3d, e), suggesting a possible involvement of Drp1 in mitochondrial fission induced by LPS, confirming observations made by others [31]. Figure 3d and e shows the influence of C_60_–COOH on LPS-induced translocation of Drp1, which indicated that C_60_–COOH notably inhibited LPS-induced translocation of Drp1. Meanwhile, BV-2 cells pretreated with C_60_–COOH showed increased phosphorylation of Drp1 at Serine637 compared with LPS-treated control cells (Fig. 4a); however, protein levels of total Drp1 and other mitochondrial fission and fusion factors, such as Fis1, Mfn1, Mfn2, and Opa1, were not significantly altered either by LPS stimulation or C_60_–COOH pretreatment (Fig. 4a, b). Therefore, it was likely that the inhibitory effects of C_60_–COOH on LPS-induced mitochondrial fragmentation might be attributed to its ability to attenuate mitochondrial localization of Drp1 promoted by modulating Ser637 de-phosphorylation. In our previous studies, we showed that C_60_ derivatives such as polyhydroxylated C_60_ derivatives regulate Nrf2 activation possibly by ROS-mediated modification of sulfhydryl groups of Keap1 [11]. However, the molecular mechanisms of regulating de-phosphorylation of Drp1 at Serine637 by C_60_–COOH still have to be elucidated.

### C_60_–COOH Inhibited LPS-Induced Mitochondrial ROS Generation and Membrane Potential Collapse (Δ*Ψ*m)

**Fig. 5:**
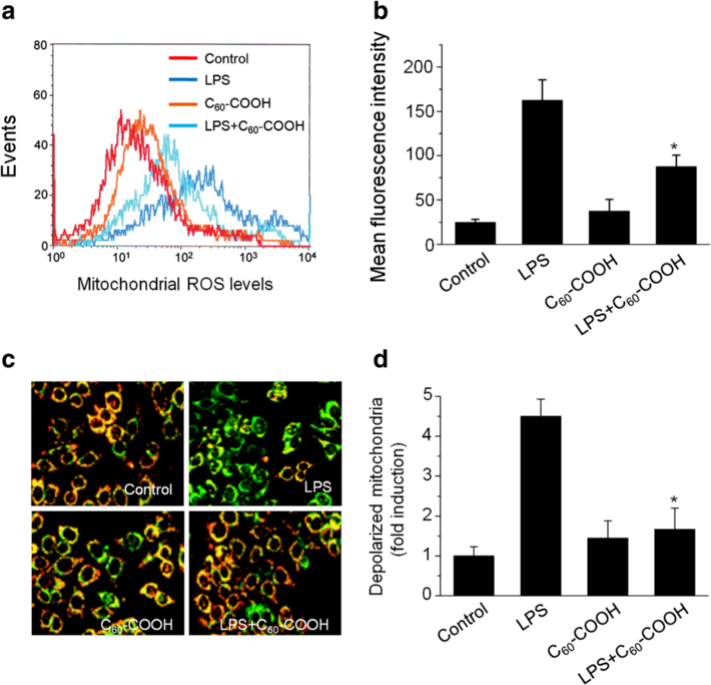
C_60_–COOH prevents loss of mitochondrial membrane potential and generation of mitochondrial reactive oxygen species (ROS) induced by LPS. BV-2 cells were treated with 1 μg/mL of LPS for 6 h in the absence or presence of 50 μM C_60_–COOH pretreatment for 6 h. The cells were then incubated either with MitoSOX Red and JC-1 fluorescent probes, respectively, and mitochondrial ROS levels and mitochondrial membrane potential were observed under a laser confocal microscope (**a** and **c**) or analyzed by flow cytometry (**b** and **d**). Representative images are shown. *p < 0.05, significantly different from LPS-treated control

Mitochondria are the primary energy producers of the cell and play key roles in cellular signaling, apoptosis, and reactive oxygen species (ROS) production [36]. Emerging evidence has linked mitochondrial dysfunction such as mitochondria fission to a variety of oxidative stress-related diseases, including neurodegenerative diseases and cancer [37]. The results shown above indicate that C_60_–COOH prevented LPS-induced increased mitochondrial fragmentation and expression of mitochondrial fission proteins. Excessive mitochondria fission events are normally associated with ROS generation and mitochondria dysfunction [32]. Therefore, we determined mitochondrial ROS level and mitochondrial membrane potential (Δ*Ψ*m) as parameters of mitochondrial fission in BV-2 cells, which were incubated with LPS with or without C_60_–COOH pretreatment by flow cytometry using MitoSOX, respectively. The results showed that the pretreatment with C_60_–COOH suppressed the LPS-mediated mitochondrial ROS generation (Fig. 5a, b) and mitochondria membrane depolarization (Fig. 5c, d). This may be attributed to the antioxidant properties of C_60_–COOH as reported earlier [10, 14]. Radical-scavenging abilities of C_60_ derivatives have been attributed to the molecular properties, including large electron affinity and formation of electron-deficient areas on the C_60_, and these properties of C_60_ derivatives may lead to direct ROS scavenging similar to that catalyzed by superoxide dismutase (SOD) [10, 14]. In addition, water-soluble C_60_ derivatives such as polyhydroxylated C_60_ (C_60_–OH) have also been reported to induce phase II antioxidant enzymes, such as heme oxygenase-1 (HO-1), γ-glutamate cysteine ligase (γ-GCS), and NAD(P)H: quinine oxidoreductase 1 (NQO-1), to attenuate oxidative stress-induced apoptosis [11]. Further studies are needed to elucidate the molecular mechanisms of C_60_–COOH to combat the deleterious action of ROS.

### C_60_–COOH Attenuated LPS-Induced Activation of NF-κB and MAPK Pathways

**Fig. 6:**
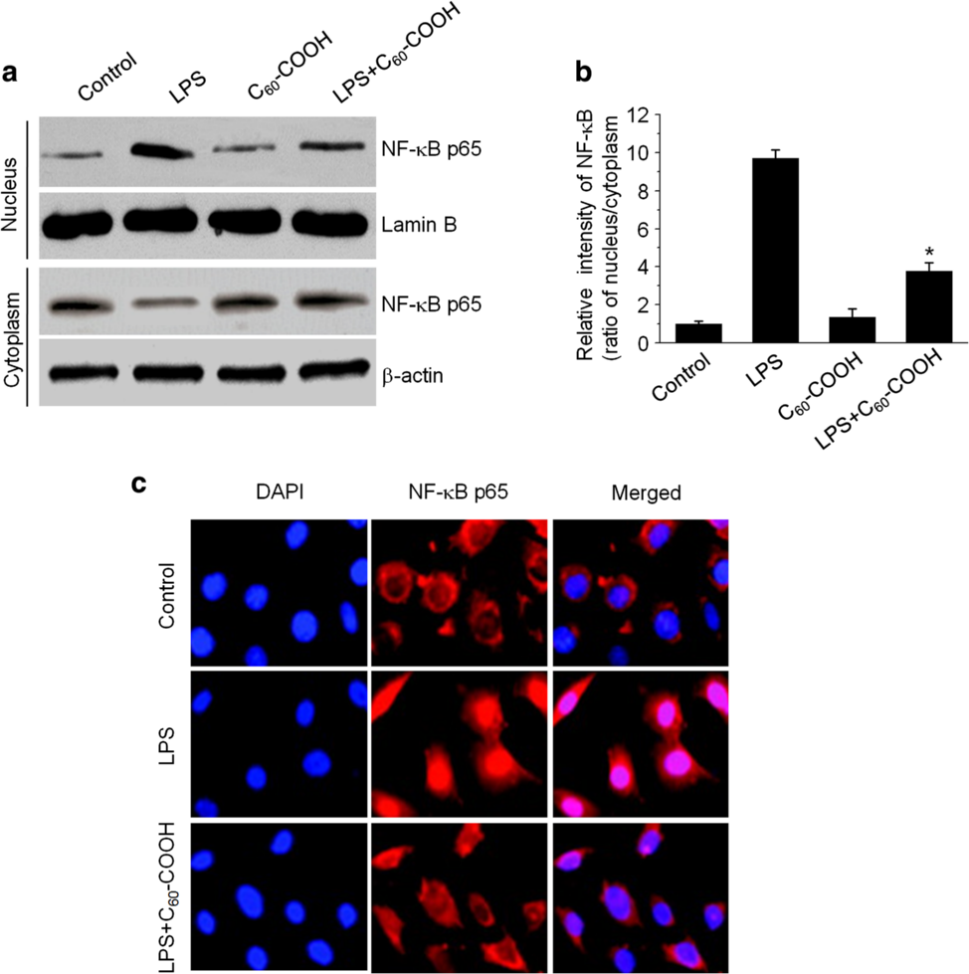
**a** Cytoplasmic and nuclear fractions from BV-2 cells treated with 1 μg/mL of LPS for 6 h with or without 50 μM C_60_–COOH pretreatment for 6 h were analyzed by western blotting using antibodies against NF-κB p65. Lamin B and β-actin were used as nucleus and cytoplasmic marker, respectively. **b** The ratio of NF-κB p65 in nucleus and cytoplasm was performed by densitometric analysis. The data are expressed as the mean ± SD of three independent experiments. *p < 0.05, significantly different from LPS-treated control. **c** Immunolocalization of NF-κB p65 was studied by confocal laser scanning microscopy

**Fig. 7:**
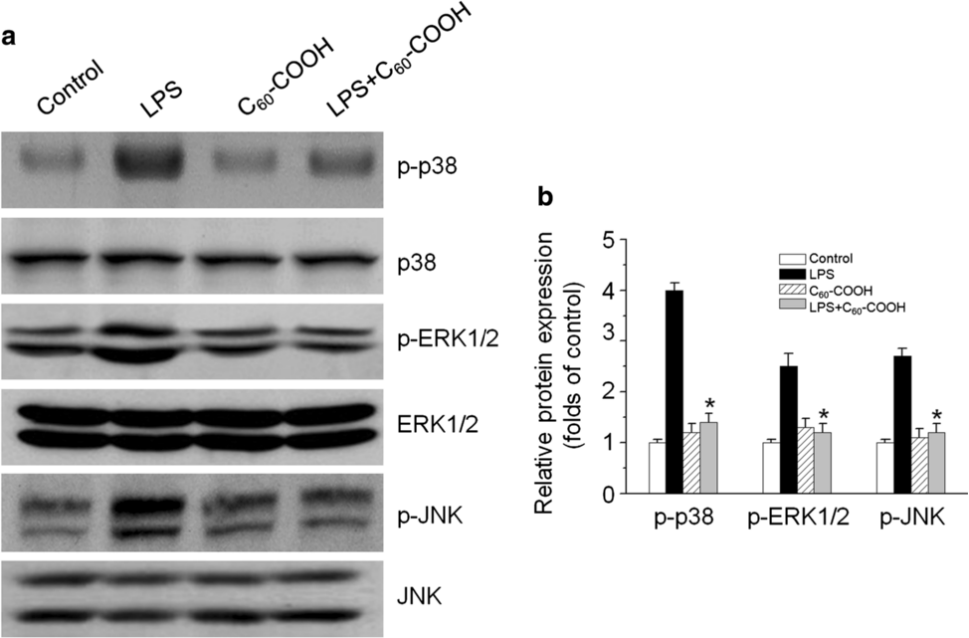
**a** BV-2 cells were treated with 1 μg/mL of LPS for 1 h with or without C_60_–COOH (50 μM) pretreatment for 6 h, and cell lysates were prepared and subjected to western blotting analysis for phosphorylated and total p38, ERK1/2, and JNK protein expression. **b** The relative levels of phosphorylated p38, ERK1/2, and JNK expression were performed by densitometric analysis. The data are expressed as the mean ± SD of three independent experiments. *p < 0.05, significantly different from LPS-treated control

Recent studies showed that mitochondrial ROS governing the LPS-induced pro-inflammatory response in microglia cells is associated with the activation of mitogen-activated protein kinase (MAPK) signaling pathways [38]. Other reports suggest that regulation of LPS-induced mitochondrial ROS is involved in the production of pro-inflammatory mediators in BV-2 microglia cells via NF-κB activation [39]. We, therefore, investigated the effect of C_60_–COOH on LPS-induced activation of NF-κB and MAPK pathways, which have been reported to associate with excessive mitochondria fission induced by LPS. Western blot analysis of nuclear and cytoplasmic proteins showed that NF-κB p65 translocation from the cytosol to the nucleus was increased in LPS-stimulated BV-2 cells, whereas pretreatment with C_60_–COOH inhibited NF-κB p65 nuclear localization (Fig. 6a,b). The nuclear translocation of NF-κB p65 from cytosol was confirmed by immunolocalization of anti-NF-κB p65 antibody using confocal microscopy (Fig. 6c). In addition, we examined whether inhibition of mitochondria fission by C_60_–COOH regulated MAPK signal pathways. Our data showed that C_60_ significantly inhibited p38 MAPK, ERK1/2, and JNK activation induced by LPS (Fig. 7a, b). This finding is similar to previous studies demonstrating that the inhibition of increasing mitochondrial fission by Mdivi-1, a mitochondrial fission inhibitor, prevents LPS-induced NF-κB and MAPK activation [32]. Our results supported the notion that mitochondrial ROS actively participate in microglia-mediated pathogenesis by altering MAPK kinase cascades and activating transcription factors NF-κB in microglia cells. Neutralization of mitochondrial ROS or suppression of the redox pathway is thus likely to alleviate inflammation. Therefore, findings from the literature along with our data imply a tight association and interplay between mitochondrial fission and ROS-mediated activation of NF-κB and MAPK signaling pathways.

### C_60_–COOH Suppressed LPS-Induced Pro-inflammatory Mediators in BV-2 Cells

**Fig. 8:**
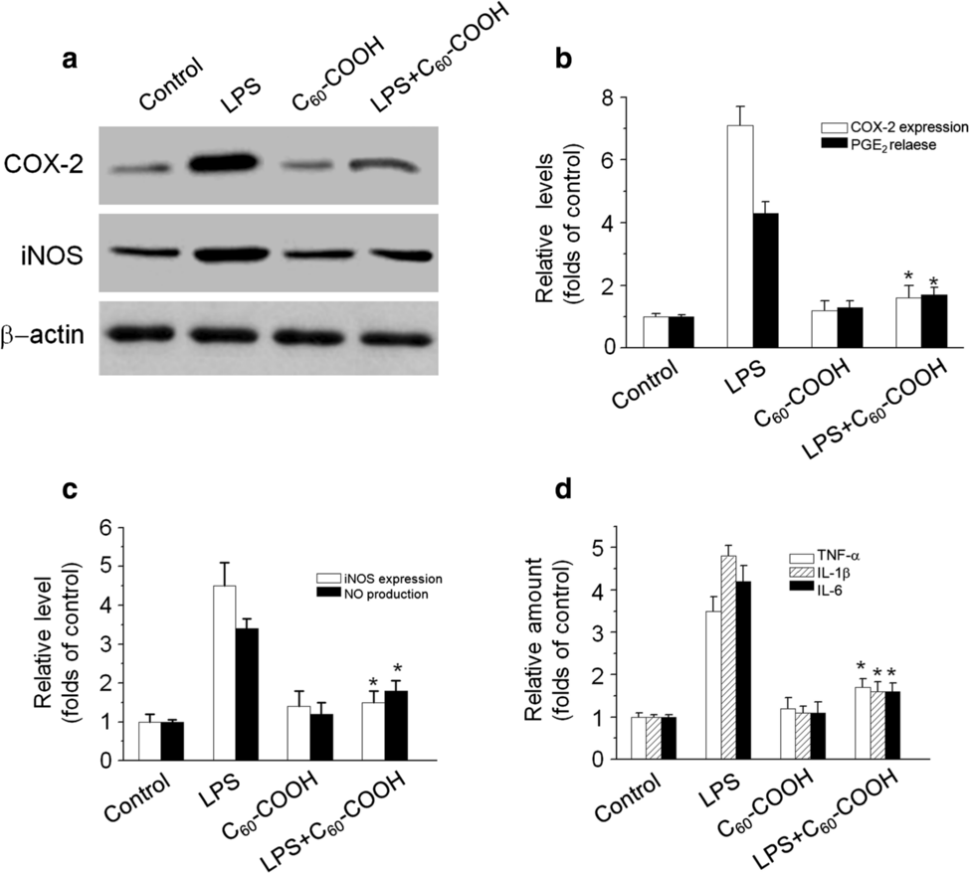
**a** COX-2 and iNOS expression were analyzed by western blotting analysis in BV-2 cells treated with 1 μg/mL of LPS for 12 h in the absence or presence of pretreatment with 50 μM C_60_–COOH for 6 h. **b**, **c** The relative levels of COX-2 and iNOS expression were performed by densitometric analysis. The data are expressed as the mean ± SD of three independent experiments. *p < 0.05, significantly different from LPS-treated control. **b**, **d** BV-2 cells were pretreated with 50 μM C_60_–COOH for 6 h and then stimulated with 1 μg/mL LPS for 12 h. The amount of PGE_2_, NO, TNF-α, IL-β, and IL-6 released into media were measured as described in the “Methods” section. The data are expressed as the mean ± SD of three independent experiments. *p < 0.05, significantly different from LPS-treated control

Microglia cells are considered the resident macrophage-like immune cells of the brain. Although microglia provide diverse beneficial functions for neuron cells, including cellular maintenance and innate immunity, constant activation of microglia can lead to detrimental neurotoxic effects due to excessive production of cytotoxic mediators such as nitric oxide (NO), prostaglandin E2 (PGE2), and other pro-inflammatory cytokines [27]. Moreover, previous studies demonstrate that mitochondria fission in activated microglia cells may be necessary for the expression of pro-inflammatory mediators [32]. Previous studies suggested that C_60_ derivatives could penetrate cell membrane, gain access to intracellular compartments, and interact with proteins, resulting in regulation of a series of signaling pathways [11, 16]. In this study, we observed that treatment with C_60_–COOH for 6 or 12 h led to a significant regulation of fission/fusion protein. The time-period of incubation was consistent with our previous observation [11], showing that pretreatment with C_60_ derivatives for up to 6 h induced Nrf2-regulated anti-inflammatory proteins such as HO-1. In this study, BV-2 cells were stimulated with LPS in the presence or absence of C_60_–COOH pretreatment for 6 h, and then the levels of various pro-inflammatory mediators were determined. As expected, our data showed that pretreatment with C_60_–COOH decreased LPS-induced up-regulated expression of pro-inflammatory protein, such as inducible nitric oxide synthase (iNOS) and cyclooxygenase-2 (Cox-2) (Fig. 8a–c). As shown in Fig. 8d, we also observed that C_60_–COOH had similar inhibitory effects on LPS-induced other pro-inflammatory mediators, such as TNF-α, IL-1β, and IL-6. These results further supported a critical role of excessive mitochondrial fission in the induction of pro-inflammatory mediators in microglia cells as described previously [32]. Therefore, it is possible that modulation of mitochondrial dynamics may be a useful therapeutic modality for preventing neurodegenerative disorders because microglia-mediated neuroinflammation is associated with neurodegenerative processes.

## Conclusions

Mitochondrial dysfunction is associated with neuropathies, and mitochondrial dynamics are altered in neurodegenerative diseases. Thus, modulation of mitochondria dynamics should lead to the development of new therapeutic strategies for treating mitochondria-associated diseases such as neurodegenerative diseases. In this study, we identified carboxylic acid C_60_ derivatives as a potent regulator of mitochondria dynamics. Although mitochondria can be considered a cellular target for C_60_–COOH, the precise mechanism of their effect on mitochondria has not been fully elucidated. Here, we first investigated the effect of C_60_–COOH on mitochondria dynamics, showing that C_60_–COOH treatment prevents mitochondria fragmentation, depolarization of mitochondria membrane potential, as well as attenuation of NF-κB and MAPK activation, thus leading to reduced neuroinflammatory response.
